# Neutrophil Extracellular Traps, Sepsis and COVID-19 – A Tripod Stand

**DOI:** 10.3389/fimmu.2022.902206

**Published:** 2022-06-10

**Authors:** Esmeiry Ventura-Santana, Joshua R. Ninan, Caitlin M. Snyder, Emeka B. Okeke

**Affiliations:** Department of Biology, State University of New York at Fredonia, Fredonia, NY, United States

**Keywords:** cytokines, inflammation, lymphocyte, septic shock, homeostasis, acute respiratory distress syndrome, pneumonia, cytokine storm

## Abstract

The novel severe acute respiratory syndrome coronavirus 2 (SARS-CoV-2) is responsible for the current coronavirus disease 2019 (COVID-19) pandemic. Majority of COVID-19 patients have mild disease but about 20% of COVID-19 patients progress to severe disease. These patients end up in the intensive care unit (ICU) with clinical manifestations of acute respiratory distress syndrome (ARDS) and sepsis. The formation of neutrophil extracellular traps (NETs) has also been associated with severe COVID-19. Understanding of the immunopathology of COVID-19 is critical for the development of effective therapeutics. In this article, we discuss evidence indicating that severe COVID-19 has clinical presentations consistent with the definitions of viral sepsis. We highlight the role of neutrophils and NETs formation in the pathogenesis of severe COVID-19. Finally, we highlight the potential of therapies inhibiting NETs formation for the treatment of COVID-19.

## Introduction

Coronavirus disease 2019 (COVID-19) was first reported in the city of Wuhan, Hubei province in mainland China in late 2019. The disease spread rapidly around the globe and was declared a pandemic by the World Health Organization on March 11, 2020 (1). In 2021, at the peak of the surge, COVID-19 was the number one cause of death in the United States (US) surpassing heart disease and cancer with an average of more than 3000 deaths per day (2). In fact, COVID-19 has led to the biggest drop in life expectancy in the US in more than seven decades (3). The successful rollout of vaccines has significantly halted mortality from the disease in the US. However, the emergence of more virulent strains of the virus remains a public health concern. As of January 2022, COVID-19 resulted in more than 800,000 deaths in the US and more than five million deaths globally with experts suggesting the number is significantly higher (4).

The causative agent is severe acute respiratory syndrome coronavirus-2 (SARS-CoV-2), whose origin is unknown. The closest human coronavirus related to SARS-CoV-2 is SARS-CoV which caused the SARS outbreak from 2002-2004 with 79% genetic similarity (5). However, SARS-CoV-2 bears the greatest genetic similarity to bat coronavirus RaTG13, with 96% similarity (6), fueling a suspicion that the virus originated from bats.

Most patients with COVID-19 have mild disease. Roughly 20% of patients exhibit exaggerated immune responses, including a hyper-inflammatory state and cytokine storm that leads to acute respiratory distress syndrome (ARDS) and eventually resulting in multi-organ damage and death. Several clinical observations indicate that severe COVID-19 meets the criteria to be classified as viral sepsis (7).

Although the cause of aberrant host immune response in severe COVID-19 is not completely understood, accumulating evidence indicates that immune dysfunction contributes to disease severity. The adaptive immune system plays a crucial role in host defense following SARS-CoV-2 infection. Antigen presenting cells (APCs) present viral antigens to CD4+ T cells which induce robust neutralizing antibody responses by B cells (8). In addition, CD8+ Cytotoxic T lymphocytes (CTLs) produce perforins and granzymes which mediate killing of virally infected cells and are important for antiviral immunity (9). Studies have shown that severe COVID-19 is associated with significant decrease in numbers of CD4+ T cells, CD8+ T cells and B cells (10, 11). Severe SARS-CoV-2 infection is also associated with an overwhelming inflammatory phenotype (12, 13). Inflammatory CD4+ Th17 cells have been shown to mediate lung damage in COVID-19 patients (14). Likewise, innate immune cells like macrophages and neutrophils have been shown to be skewed towards an inflammatory phenotype in SARS-CoV-2 infection (15, 16). In particular, the production of neutrophil extracellular traps has been shown to propagate severe COVID-19 (17–19). The role of T and B cells in COVID-19 has been extensively reviewed (8, 20, 21) and we will focus on the role of neutrophils in the pathology of severe COVID-19.

In this article, we highlight important observations which indicate that severe COVID-19 has clinical presentations consistent with the definitions of viral sepsis. We discuss the significant contribution of neutrophils in driving disease pathology following infection with SARS-CoV-2 *via* formation of neutrophil extracellular traps (NETs). Furthermore, we highlight the potential of therapies inhibiting NETs formation for the treatment of severe COVID-19.

## NETs and Inflammation

Polymorphonuclear neutrophils (PMNs) are the most abundant white blood cells in circulation and are rapidly deployed to the site of bacterial, fungal or viral infection as a critical part of host defense (22, 23). The role of neutrophils in host defense is widely appreciated and defective neutrophil function is associated with recurrent infections or occurrence of rare diseases (24). For several decades, neutrophils have been known to kill pathogens through phagocytosis and oxidative burst accompanied by granular release of potent antimicrobials (25). Recently, neutrophils were shown to kill microbes through the release of neutrophil extracellular traps (NETs). NETs are web-like extrusions, composed of a DNA framework and decorated with granular proteins like neutrophil elastase (NE) and myeloperoxidase (MPO) (26).

The molecular mechanisms involved in NET formation is incompletely understood and the processes that lead to the release of DNA by neutrophils is still a subject of debate. It has been reported that neutrophils form NETs through a tightly regulated cell death pathway called NETosis that involves collapse of the nuclear envelope and rupture of the cytoplasmic membrane (27). Studies have also shown that neutrophils release NETs in the absence of cell death (28, 29). These discrepancies may be due to the use of different stimulants for NET induction. Nevertheless, the critical role of certain enzymes and molecules in NET formation including NE, NADPH oxidase complex, peptidylarginine deiminase 4 (PAD4) and the protein kinase C (PKC) pathway have been highlighted and reviewed elsewhere (30–32). NETs have been shown to kill bacteria, fungi, viruses, and parasites (26, 33–35) and there is significant interest in the role of NETs in SARS-CoV-2 infection.

Although NET formation is a mechanism of host defense, excessive NET formation or defective clearance of NETs triggers sustained inflammatory response that can lead to organ damage and drive disease pathology. For example, histones released during NET formation have been shown to be cytotoxic and damage endothelial cells (36). NET formation leads to the production of autoantibodies that damage important organs (37) and inhibition of NETs formation has been shown to be protective in several models of inflammatory diseases (38, 39). Accumulating evidence indicates that NETs contributes to the pathophysiology of severe COVID-19 (18, 19). The role of NETs in the pathophysiology of COVID-19 constitutes a major focus of this review and will be discussed in later sections.

## Viral Sepsis

Despite decades of research and treatment, sepsis still constitutes a major challenge in modern medicine and is a leading cause of death in the intensive care unit (ICU). Sepsis is a heterogeneous and dynamic syndrome, due to a complex interplay between the host immune response and the invading microbe. The Third International Consensus Definitions Task Force defined sepsis as life-threatening organ dysfunction caused by a dysregulated host response to infection (40). This definition implies the general notion that bacteria, fungi and viruses can equally cause sepsis. However, there has been concerns that physicians are reluctant to designate viral infections as a case of sepsis (7). Although, bacteria accounts for more than 70% of documented sepsis (41, 42), the role of viruses in sepsis should not be ignored and this knowledge is important to tailor adequate treatment to culture negative patients.

The global burden of viral sepsis is huge with an estimated occurrence of 200 million cases of viral community-acquired pneumonia (CAP) each year (43). Pneumonia is the most common clinical syndrome in patients with sepsis (41, 42). Interestingly, studies have shown that viruses are the most common causes of CAP (44, 45). Therefore, the strict association of sepsis with bacterial infection can be costly given that early antiviral therapy is associated with better outcome in viral sepsis (46). It is also concerning that antibiotics have been administered in culture negative cases of pneumonia (47) indicating the bias of physicians to ignore viruses as a veritable cause of sepsis. It must be stated that the presence of a virus is not sufficient for the diagnosis of viral sepsis. This is due to the possibility of bacterial co-infection or bacterial sepsis resulting from virus-induced immunosuppression. However, among patients with a diagnosis of pure viral CAP, 61% and 7% presented with sepsis and septic shock respectively upon admission to the clinic (47).

Several viruses have been reported to cause sepsis including influenza viruses, rhinoviruses, respiratory syncytial viruses, adenoviruses, herpes simplex viruses, human enteroviruses, dengue viruses and coronaviruses (7, 47). Importantly, the betacoronaviruses – Middle East respiratory syndrome coronavirus (MERS-CoV), SARS-CoV, and SARS-CoV-2 that threaten global health have also been known to cause sepsis. For example, patients with severe COVID-19 have clinical symptoms of viral sepsis. In one study, 59% of patients with COVID-19 were diagnosed with sepsis (48). Importantly, 76% of COVID-19 patients diagnosed with sepsis were negative for bacterial or fungal infections (48). Another study diagnosed sepsis in 100% of patients who died of COVID-19 (49). More studies are required for the diagnosis of sepsis in critically ill patients with COVID-19. However, taking into consideration several clinical observations and the above definition of sepsis, the authors agree that severe COVID-19 is a typical case of dysregulated host response to infection and therefore qualifies as sepsis caused by SARS-CoV-2 infection.

## Pathophysiology of Sepsis

The normal immune response to microbial invasion leads to the activation of host defense mechanisms to counter the microbe and prevent colonization of the host by the microbe. This involves cellular activation, vasodilation, leukocyte recruitment and increased endothelial permeability (50, 51). This complex and well-choreographed mechanism of immune activation describes the inflammatory response. Overwhelming infection caused by a virulent microbe or dysregulated immune response to an infection can lead to an overtly exaggerated immune activation or hyper-inflammatory state causing tissue injury and collateral damage to the host.

Innate immune cells like neutrophils and macrophages express molecular receptors called pattern recognition receptors (PRRs) that recognize pathogen-associated molecular patterns (PAMPs) on microbes (52). Several PRRs have been described and among them, TLRs are the most studied.

SARS-CoV-2 is an enveloped virus, with a single-stranded, positive-sense RNA genome (53). During replication, RNA viruses produce double-stranded RNA (dsRNA) as an intermediate (53). Both ssRNA and dsRNA can activate TLRs leading to the production of proinflammatory cytokines *via* MyD88 and NFk-B activation (54, 55).

Innate immune cells also play a role in the maintenance of antiviral state by the activation of Stimulator of Interferon Genes (STING) pathway (56–58). Upon activation, STING recruits TANK binding kinase 1 (TBK1) and the STING-TBK1 complex subsequently phosphorylates Interferon Regulatory Factor 3 (IRF3) (58). STING can also stimulate IKK leading to NF-κB activation (58). The transcription factors, IRF3 and NF-κB induce the production of type I IFNs and other pro-inflammatory cytokines important for antiviral immunity (58). For example, activation of STING pathway has been shown to block human coronavirus infection (59) and defective type I IFN production is associated with severe COVID-19 (60, 61).

The production of cytokines *via* NF-κB activation is an important step for the recruitment of neutrophils and other immune cells. However, a major hallmark of sepsis and severe COVID-19 is the excessive production of pro-inflammatory cytokines termed cytokine storm (CS) (7, 49). Cytokines like tumor necrosis factor (TNF), Interleukin (IL)-1, IL-6, IL-8, IL-12 and IL-17 propagate the inflammatory response through leukocyte recruitment, release of secondary inflammatory mediators, endothelial dysfunction and NETs formation (7, 18). For example, TNF and IL-1 induce vasodilation, facilitate the release of secondary mediators such as nitric oxide (NO), platelet activation factor (PAF), prostaglandins, leukotrienes and the activation of the complement system (62). Indeed, CS has been implicated in the pathogenesis of sepsis, viral diseases, autoimmune diseases, cancer and COVID-19 (18, 62–65).

CS also promotes leukocyte recruitment and endothelial permeability in the pulmonary capillaries resulting in lung injury and acute respiratory distress syndrome (ARDS) (64). Microbes associated with pulmonary infection will induce neutrophil migration to the lungs. The lumen of the pulmonary capillaries are more narrow and this leads to extended transit time along the pulmonary endothelium. Neutrophil accumulation and sequestration in the lungs leads to prolonged release of proteolytic enzymes that results in acute lung injury (ALI) and ARDS (66). Sepsis is the most common cause of ARDS and sepsis-related ARDS is associated with overall higher disease severity, longer ICU stays and mortality (67, 68).

Additionally, cytokine activity also activates the coagulation pathway, which can lead to disseminated intravascular coagulation (DIC) and/or coagulopathy which is a hallmark of sepsis (62). Aberrant activation of the coagulation pathway leads to capillary microthrombi, tissue hypoperfusion and end-organ ischemia (69).

Overall, there is consensus that sepsis is driven by the host immune response to infection rather than the pathogen itself (63). However, several clinical trials of therapies targeting important steps in the host immune response during sepsis have not been successful (62). We anticipate that advances in technology will increase our knowledge of sepsis pathogenesis leading to more novel therapeutic interventions.

## NETs, Sepsis and Severe COVID-19

Neutrophils are the first immune cells to arrive at the site of bacterial infection and play an important role in host defense. These cells are equipped with antimicrobial granular content that is rapidly deployed to eliminate the invading microbe. However, there is unequivocal experimental evidence that neutrophils contribute to sepsis pathology by release of cytolytic granular content, vaso-occlusion, and NET formation (66, 70).

The discovery of the process of NET formation by neutrophils highlighted a novel mechanism of innate immune defense against microbes. NETs have been shown to trap and kill a wide range of microbes including bacteria, fungi and viruses (26, 33–35). NETs formation can be beneficial during sepsis because NETs spatially restrict the dissemination of microbes during infection (26). To prevent physical containment by NETs, some bacteria have evolved to degrade NETs and NET degradation promotes bacterial virulence (71). Patients with chronic granulomatous disease (CGD) caused by mutations in genes encoding NADPH oxidase subunits do not make NETs and are susceptible to recurrent life-threatening infections (72). Gene therapy in a CGD patient restored NET forming ability of neutrophils resulting in clearance of refractory fungal infection (72). Additionally, NET proteins like histones, NE, MPO and proteinase 3 (PR3) have potent antimicrobial properties and help in bacterial killing (73).

However, accumulating evidence suggests that NETs formation is a double-edged sword (74) that contributes to the pathogenesis of several diseases including sepsis (70), rheumatoid arthritis (75), vasculitis (76), diabetes (77), lupus (78), cancer (79) and COVID-19 (18, 80). For example, studies have shown that levels of circulating cell-free DNA that are released during NET formation is a strong predictor of sepsis mortality (81). Also, histones which are the most abundant proteins in NETs (82) are cytotoxic towards epithelial and endothelial cells (36, 83). Histone administration to mice resulted in neutrophil accumulation in the lungs, microvascular thrombosis and death (83). Additionally, in non-human primates challenged with lethal concentration of *E. coli*, histone levels correlate with onset of renal failure. Furthermore, using three different models of sepsis: injection of LPS, injection of TNF, and CLP, the authors showed that antibodies against H4 improved animal survival (83). Consistent with this, we recently showed that inhibition of NE produced during NET formation reduced lung neutrophil accumulation, systemic levels of proinflammatory cytokines and improved survival in a mouse model of endotoxic shock (38).

A major complication attributed to NETs formation is thrombosis resulting in multi-organ failure (84–87). Due to their ability to form scaffolds, NETs can occlude blood vessels and cause thrombosis. NET scaffolds also promote adhesion of platelets leading to thrombus formation (85, 86). Importantly, serine proteases released by NETs like neutrophil elastase enhance tissue factor and factor XII dependent coagulation thereby leading to intravascular thrombus formation (88). Histones produced by NETs can promote platelet aggregation and thrombin generation *via* toll-like receptor (TLR) 2 and 4 (89). Interestingly, activated platelets have been shown to induce *de novo* NETs formation thereby propagating the vicious circle of platelet-neutrophil interaction in coagulopathy (90–92). Indeed, dysregulated NETs formation is associated with coagulopathy in sepsis and severe COVID-19 (80, 92–95).

We have drawn comparisons between sepsis and severe COVID-19 and conclude that the clinical presentations of sepsis and severe COVID-19 intersect at so many levels. Sepsis and severe COVID-19 commonly affect the pulmonary, cardiovascular, and renal systems. Many patients with severe COVID-19 exhibited clinical manifestations of shock-like cold extremities, weak peripheral pulses, dysfunction in microcirculation and organ damage notably in the lungs, kidney and liver (96). Like sepsis, ARDS and respiratory failure is the most common cause of death in COVID-19 patients (49, 97). Additionally, like sepsis, mortality in severe COVID-19 is driven by risk factors like age and presence of predisposing conditions (49). Severe COVID-19 is also characterized by excessive inflammatory cytokine production (19, 98, 99). Moreover, C-reactive protein, a biomarker for sepsis severity has also been shown to predict poor prognosis in COVID-19 (100). Furthermore, similar to sepsis, patients with severe COVID-19 show evidence of coagulopathy and dysregulated thrombus formation (80, 101). Indeed, one study showed that 100% of patients who died from COVID-19 were diagnosed with sepsis (49). In line with the evidence given above, we argue that severe COVID-19 is a typical case of viral sepsis.

**Figure 1:**
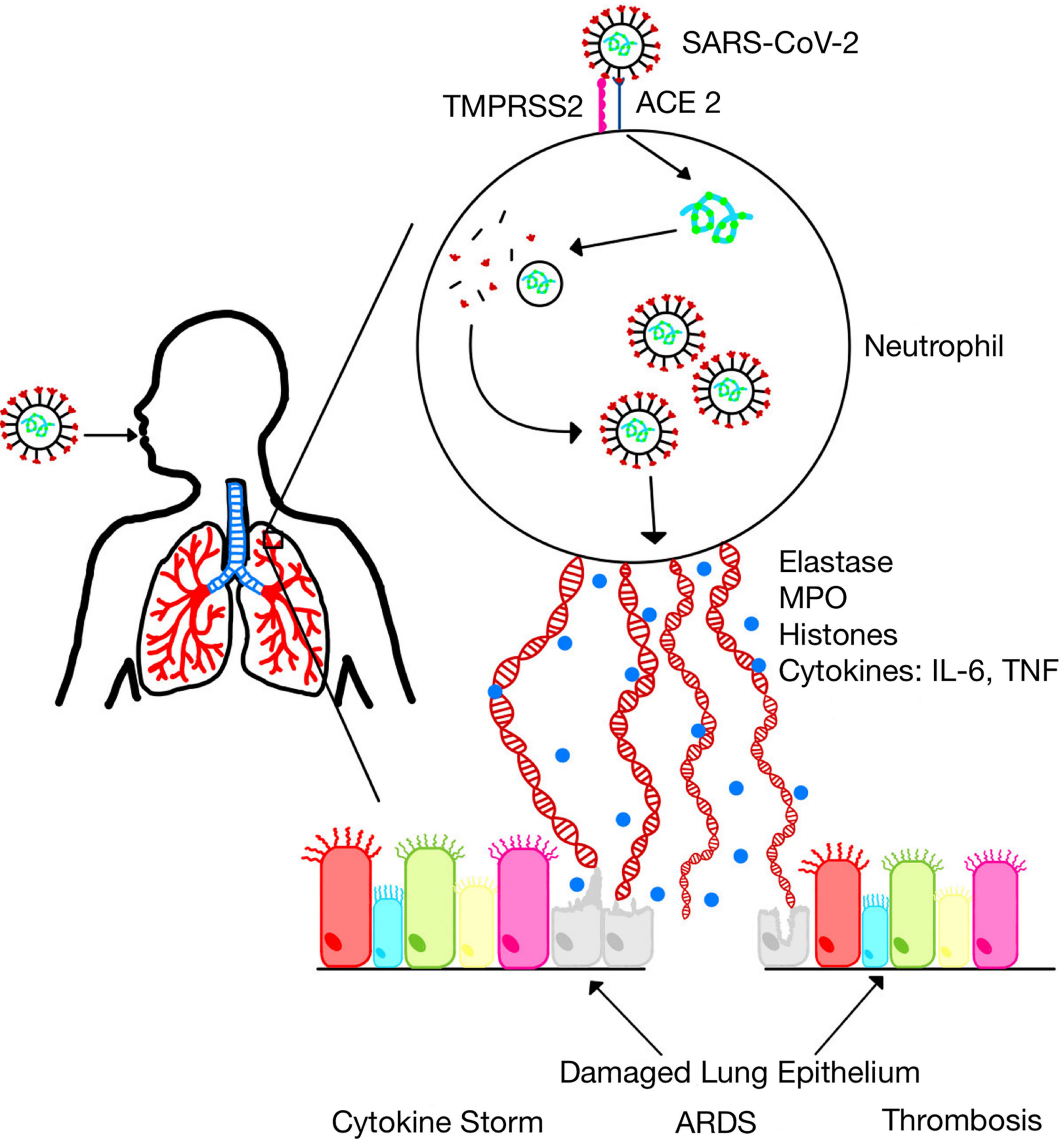
SARS-CoV-2 infection induces neutrophil extracellular traps. SARS-CoV-2 replicates in neutrophils and induces the formation of NETs which leads to the release of inflammatory cytokines and several proteins that damage lung epithelium resulting in acute lung injury and acute respiratory distress syndrome (ARDS).

Since NETs have been shown to contribute to sepsis pathology, it is conceivable that NETs may contribute to the pathogenesis of severe COVID-19 (**Figure 1**). Indeed, several studies have implicated NETs in the pathogenesis of severe COVID-19. For example, it was shown that SARS-CoV-2 replicates in neutrophils and triggers NETosis which contributes to COVID-19 pathology by killing lung epithelial cells (102). Sera from patients with COVID-19 have elevated levels of markers of NET formation including cell-free DNA, MPO-DNA, citrullinated histone H3, and neutrophil elastase (**Table 1**) and these markers are associated with disease severity (18, 111–113). Neutrophilia and NETosis is a major cause of ARDS and lung injury in severe COVID-19 (80, 114, 115). NETs formation is associated with systemic inflammation and cytokine storm which contributes to mortality in severe COVID-19 (116, 117). Additionally, dysregulated thrombus formation which contributes to mortality in severe COVID-19 has been associated with NET formation (80, 118, 119). Furthermore, COVID-19 has been shown to induce the production of autoantibodies associated with NET production (101). These observations have led to an overwhelming scientific support for targeting NETs formation as a veritable approach for the treatment of severe COVID-19 (19, 120, 121).

**Table 1:** NET proteins associated with severe COVID-19.

NET COMPONENT	REFERENCE
DNA	(18, 103)
Elastase	(104)
Myeloperoxidase	(18, 105)
Proteinase 3	(105, 106)
Histone 3	(18, 103)
Cathepsin G	(104, 107)
Azurocidin	(108)
Transketolase	(104, 109)
Alpha-defensins	(110)
Calprotectin	(110)

## Targeting NETs in COVID-19

Recently, there has been concerted efforts to develop therapies targeting NETs in several diseases. Therapies targeting NETs have shown excellent success in mitigating lung inflammation and ARDS in preclinical models (19, 38). Since ARDS is the major cause of death in COVID-19, we advocate for the investigation of NET therapies in the treatment of COVID-19 patients. Different approaches to targeting NETs have shown remarkable success in preclinical models. Such approaches include dissolving NET backbone, for example using DNAse (122), blocking molecules relevant in NET formation for example ROS, PAD4 and gasdermin D (39, 123) or blocking the activity of NET proteins like neutrophil elastase (38). Some of these NET therapeutics are currently available in the clinic and should be considered for the treatment of patients critically ill with COVID-19. For example, DNAse treatment is used in the clinic for patients with cystic fibrosis and the NE inhibitor sivelestat is clinically approved for the treatment of ARDS in Japan and South Korea (124, 125). Indeed, clinical trials of several NET inhibitors are underway for the treatment of COVID-19 (**Table 2**) and some of them have already been adopted as the standard of care for COVID-19 patients. For example, glucocorticoid therapy which is one of the earliest anti-inflammatory treatments available for sepsis patients has been shown to be beneficial for COVID-19 patients and dexamethasone is routinely given to COVID-19 patients (133). Importantly, dexamethasone has been shown to reduce NETs formation (134). Heparin, another NET inhibitor has also been shown to be beneficial for the treatment of COVID-19 patients (135, 136).

**Table 2:** Clinical trials of NET inhibitors for COVID-19 Treatment.

NET INHIBITOR	MOLECULAR TARGET/FUNCTION	COVID TRIAL
Pulmozyme (dornase alfa) (126)	Recombinant DNase that improves lung function by thinning sputum.	NCT04359654 NCT04409925
Brensocatib (127)	Inhibits dipeptidyl peptidase 1 and neutrophil proteases	NCT04817332
Anakinra (128)	Interleukin-1 Receptor antagonist	NCT04594356
Glucocorticoid (methylprednisolone) (129)	Immunosuppressive treatment	NCT04244591
Hydroxychloroquine (130)	Reduces activity of immune system by disrupting lysosomal stability	NCT04332991
Colchicine (131)	Anti-inflammatory	NCT04326790
Alvelestat (132)	Neutrophil Elastase inhibitor	NCT00769119

Anti-inflammatory therapies and anti-cytokine therapies can also be beneficial in reducing neutrophilia, NETs formation and NET-induced thrombosis. For example, elevated levels of IL-6 has been associated with severe COVID-19 thereby highlighting IL-6 as a therapeutic target. IL-6 signaling has been shown to promote NET formation and lung inflammation (137). We recently showed that inhibition of NETs formation led to decrease in systemic levels of IL-6 and improved survival in a mouse model of endotoxic shock (38). Indeed, Tocilizumab, a recombinant humanized monoclonal anti-IL-6 antibody targeting the human IL-6 receptor was recently approved for the treatment of COVID-19 (138). Previous studies showed that Tocilizumab is also associated with decrease in NET formation (139).

As our understanding of the molecular mechanisms of NET formation increases, more therapies targeting NETs will become available and may hold promise for the effective treatment of severe COVID-19.

## Concluding Remarks

The management of sepsis has been a challenge in modern medicine and the launch of surviving sepsis campaign was aimed to curtail the unacceptably high mortality of sepsis patients in the ICU (40). The mortality induced by the novel SARS-CoV-2 responsible for the current global pandemic has been attributed to sepsis (49). In this regard, biomarkers used for sepsis can be used for the early identification of COVID-19 patients that are at risk of progressing to severe disease. There is consensus that mortality in sepsis and COVID-19 is due to host immune response. Hence, modulating dysfunctional immune response in COVID-19 is critical for improving survival.

The formation of neutrophil extracellular traps has emerged as a contributing factor to the pathogenesis of COVID-19 (102). Importantly, SARS-CoV-2 has been shown to infect neutrophils and promote NETosis (102). Understanding of the role of NETs in the pathogenesis of severe COVID-19 holds potential for improving survival of patients. NET biomarkers can be easily detected in the blood and has been shown to indicate disease severity in COVID-19 (18). Hence, biomarkers of NET formation can be used to stratify COVID-19 patients at risk of progressing to severe disease. Since therapies targeting NETs have shown success in experimental models of ARDS, we propose that therapies targeting NETs have great potential for the treatment of COVID-19.

While we have focused on the role of extracellular traps produced by neutrophils in this review, macrophages also produce macrophage extracellular traps (METs) which propagate inflammation (140, 141). Interestingly, macrophages have been shown to contribute to inflammation in COVID-19 (12). Moreover, neutrophil extracellular traps from COVID-19 patients induce a proinflammatory response in monocyte-derived macrophages thereby linking NET formation to inflammatory macrophage activity (17). It is worthy of note that similar to neutrophils, macrophages also release elastase, histones and MPO during MET formation (142, 143). Hence, it is conceivable that mechanisms inhibiting the formation of NETs as highlighted here will also inhibit the formation of METs. Studies investigating the role of macrophage extracellular traps in severe COVID-19 will help unravel its role in the condition.

As with the case in sepsis, it is likely that one drug may not be sufficient to improve survival in COVID-19. Rather, a combinatorial approach may be necessary to reverse mortality in COVID-19. For example, the recently approved Tocilizumab showed benefit for COVID-19 patients who received it in conjunction with corticosteroids (144). We advocate for clinical trials investigating such combinations of NET therapeutics for the treatment of COVID-19. As another example, although sivelestat did not improve mortality in patients with ARDS (145), combination of sivelestat with antiviral therapy or another NET inhibitor may be beneficial for COVID-19 patients.

The intelligent design of clinical trials of therapies targeting NETs is essential and several factors including timing of intervention is critical for success. For example, there are concerns that DNase may enhance the dispersal of free histones and promote inflammation thereby leading to worse outcome in sepsis. In line with this, Meng et al, showed that early administration of DNase led to hyper-susceptibility to polymicrobial sepsis in mice (146). In a follow-up study, Mai et al showed that delayed administration of DNase is necessary for improved outcome in sepsis (147).

More research is needed to understand neutrophil behavior during SARS-CoV-2 infection. For example, an interesting question is whether different viral strains that have varying degrees of immunogenicity differ in their degree of NET induction, and this remains an important subject of investigation in our laboratory. Increase in our knowledge and understanding of the pathogenesis of COVID-19 will widen the availability of molecular targets that will yield the desired therapeutic benefit. With concerted research efforts, the menace of severe COVID-19 in the ICU will be curtailed.

## Author Contributions

EV did literature search and wrote portions of the manuscript. JN also did literature search and wrote portions of the manuscript. EO corrected and edited the manuscript for publication. CS did literature review and table for manuscript revision. All authors contributed to the article and approved the submitted version.

## Funding

Funding for this work was provided by the State University of New York at Fredonia.

## Conflict of Interest

The authors declare that the research was conducted in the absence of any commercial or financial relationships that could be construed as a potential conflict of interest.

## Publisher’s Note

All claims expressed in this article are solely those of the authors and do not necessarily represent those of their affiliated organizations, or those of the publisher, the editors and the reviewers. Any product that may be evaluated in this article, or claim that may be made by its manufacturer, is not guaranteed or endorsed by the publisher.
